# Analytical Validation of a Pan-Cancer Panel for Cell-Free Assay for the Detection of *EGFR* Mutations

**DOI:** 10.3390/diagnostics11061022

**Published:** 2021-06-02

**Authors:** Min-Kyung So, Jong-Ho Park, Jong-Won Kim, Ja-Hyun Jang

**Affiliations:** Department of Laboratory Medicine and Genetics, Samsung Medical Center, School of Medicine, Sungkyunkwan University, Seoul 06351, Korea; mkso79@gmail.com (M.-K.S.); parkjhozz@gmail.com (J.-H.P.); culture.jkim@gmail.com (J.-W.K.)

**Keywords:** gene panel, liquid biopsy, circulating tumor DNA

## Abstract

Liquid biopsies have increasingly shown clinical utility. Although next-generation sequencing has been widely used for the detection of somatic mutations from plasma, performance characteristics vary by platform. Therefore, thorough validation is mandatory for clinical use. This study aimed to evaluate the analytical validity of the Oncomine Pan-Cancer Cell-Free Assay. A massively parallel sequencing for the assay was performed using the Ion S5 XL System with Ion 540 kit. The analytical sensitivity and precision were evaluated using pre-characterized reference materials. The specificity was evaluated using plasma from healthy subjects. A comparison with the Cobas EGFR Mutation Test v2 was performed using reference materials and plasma from lung cancer patients. For SNVs and short indels, the analytical sensitivities at variant allele frequencies (VAFs) of 0.1%, 0.5%, and 1% were 50%, 93.4%, and 100% with 20 ng of input, respectively. The overall precision of the true positive variants was 98% at a VAF of 1% with 20 ng input. The assay showed a similar sensitivity to that of the Cobas EGFR Mutation Test v2 at a VAF of 0.5% with 20 ng of input and 100% concordance on clinical samples. The Pan-Cancer Cell-Free Assay can be applied to detect *EGFR* mutations in advanced lung cancer patients, although follow-up studies will be needed to evaluate the analytical validity for other types of genes and aberrations using clinical samples.

## 1. Introduction

Cell-free DNA (cfDNA) is a short-fragment nucleotide in the bloodstream. This DNA is produced from cell apoptosis or necrosis but is also actively released from viable cells [1,2,3]. Circulating tumor DNA (ctDNA), as a component of cfDNA of cancer patients, represents the mutation profile of tumor tissues, and the concordance between tissue and plasma ctDNA ranges from ~70% to ~90% for the detection of EGFR variants in nonsmall cell lung cancers [4,5]. Research has been conducted to demonstrate the usefulness of ctDNA in various cancer types for the monitoring of tumor recurrence, the identification of mutations for targeted therapy, and the early detection of tumors [6]. Recently, the guidelines of the American Society of Clinical Oncology for molecular testing of lung cancer patients have been updated. The guideline states that the cfDNA assay can be used to identify EGFR mutations in some clinical situations with limited and/or insufficient tissues [7].

For the detection of ctDNA, a highly sensitive methodology is essential due to the small amount of cfDNA. Furthermore, ctDNA is present in the background of cfDNA derived from hematopoietic cells [8]. Among the various methods available for analysis, real-time PCR, digital droplet PCR, and next-generation sequencing (NGS) are commonly used in clinical laboratories. As a real-time PCR technique, the Cobas EGFR Mutation Test v2 (Roche Molecular Systems, Inc., Branchburg, NJ, USA) is used for detecting several hotspot mutations of the *EGFR* gene. The test, which uses plasma, as well as formalin-fixed paraffin-embedded tissue specimens, was approved as a companion diagnostic test by the United States Food and Drug Administration [9]. Digital droplet PCR can detect 0.01~0.1% of the mutant burden at hotspot positions [6,10]. An NGS-based analysis, in contrast to a targeted approach, can include up to several hundred genes and detect sequence variations, copy number variants (CNVs), and/or fusion transcripts in a single test. Such an analysis shows a 0.1% limit of detection when using a deeper depth of coverage, special processes such as molecular barcodes (also known as unique molecular identifiers) for the differentiation of true mutations from sequencing artifacts, and computational postprocessing [8,11,12].

This study aimed to evaluate the analytical performance of the Oncomine Pan-Cancer Cell-Free assay (Thermo Fisher Scientific, Waltham, MA, USA) using cell-free total nucleic acid (cfTNA), which is designed to identify single-nucleotide variants (SNVs), small indels, copy number variations (CNVs), and RNA fusions from 52 genes. In addition, we compared the analytical sensitivity of the Oncomine Pan-Cancer Cell-Free assay (Thermo Fisher Scientific, Waltham, MA, USA) to that of the Cobas EGFR Mutation Test v2 (Roche Molecular Systems, Pleasanton, CA, USA).

## 2. Materials and Methods

### 2.1. Preparation of Pre-Characterized Reference Materials

For validation of the analytical performance, we used two commercially available control materials. For validation of SNVs and indels, the Multiplex I cfDNA Reference Standard Set (HD778 for a variant allele frequency (VAF) of 1%, HD779 for a VAF of 0.1%, and HD776 for wild type; Horizon Discovery, Waterbeach, UK) was used, which contains 4 *EGFR* mutations (p.L858R, p.E746_A750del (∆E746-A750), p.T790M, p.A767_V769dup (V769-D770insASV)), 1 *KRAS* mutation (p.G12D), 2 *NRAS* mutations (p.Q61K, p.A59T), and 1 *PIK3CA* mutation (p.E545K). In addition, a library with a VAF of 0.5% was prepared by mixing the mutant control (HD778) and wild-type control (HD776) in equal quantities. For the nucleic acid input, the control material at an initial concentration of 20 ng/μL was further diluted, and different amounts of the nucleic acid input (5, 10, and 20 ng) were used for library preparation. The HD786 structural multiplex cfDNA Reference Standard Set was used for validation of the CNV and fusion. This HD786 reference standard set contains 5% fusion of SLC34A2/ROS1 and CCDC6/RET, 4.5 copies (2.25-fold change) of *MET*, and 9.5 copies (4.75-fold change) of *MYCN*. In addition, HD786 was mixed with wild-type control at a ratio of 1:4, yielding a VAF of 1% for the fusion transcript, 2.5 copies (1.25-fold change) of *MET*, and 3.5 copies (1.75-fold change) of *MYCN*.

### 2.2. Preparation of Clinical Samples

We used plasma samples from eight lung cancer patients with *EGFR*-positive variants detected using the Cobas EGFR Mutation Test v2 (Roche Molecular Systems, Pleasanton, CA, USA). Plasma was separated from the Cell-Free DNA Collection tube (Roche Molecular Systems) by double centrifugation (1600× *g* for 15 min followed by 16,000× *g* for 10 min); after completion of the test, the residual volume was archived in a deep freezer (−70 ℃). As the residual volume of plasma of each sample was not sufficient, two *EGFR*-positive clinical samples were mixed to 4 mL of plasma for DNA extraction. Extraction of cfTNA was performed using the QIAamp^®^ DSP Circulating NA kit (Qiagen, Hilden, Germany). The concentration and size distribution of the nucleic acid were assessed using Qubit 3.0 Fluorometer (Thermo Fisher Scientific, Waltham, MA, USA) and Tapestation (Agilent, Santa Clara, CA, USA), respectively. From the 40 μL of eluted cfTNA, 10 μL (the maximum volume of input recommended by the manufacturer) was used for library preparation if the total amount of cfTNA did not exceed 50 ng. This study was approved by the Institutional Review Board of Samsung Medical Center, Seoul, Korea (2019-01-140).

### 2.3. Library Preparation, Sequencing, and Variant Calling

The Oncomine Pan-Cancer Cell-Free Assay was used for library preparation, in which DNA fragments were each tagged with a unique molecular tag. Four libraries were loaded on an Ion 540 kit using Ion Chef (Thermo Fisher Scientific, Waltham, MA, USA). One or two Ion 540 chips were sequenced simultaneously using the Ion S5 XL System (Thermo Fisher Scientific). In the initial validation, 16 libraries were sequenced during two consecutive sequencing runs; two 540 chips were analyzed at each sequencing run. In the subsequent six sequencing runs, one control material was included in each run and the corresponding data of the control material were used for validation of reproducibility. Sequence alignment to the hg19 human reference genome and variant calling were performed using Torrent Suite version 5.10.1 (Thermo Fisher Scientific). For variant annotation, Ion Reporter Software 5.10 was used (Thermo Fisher Scientific). Cutoffs for various parameters were used as the default setting. Briefly, molecular coverage, the representation of the number of DNA molecules, of at least 3 with a minimum detection cutoff frequency of 0.065% must be satisfied for variant calling for SNV/indel. For a CNV call, the following criteria must be met: median of the absolute values of all pairwise differences (MAPD) < 0.4, *p*-value < 10^−5^, and CNV ratio for a copy number gain >1.15-fold change. For calling a fusion transcript or *MET* exon 14 skipping, more than 2 molecular counts of the target must have been reported. In addition, at least one of the two control genes (*TBP* and *HMBS*) and wild-type controls of *MET* must have a molecular count above 2. A variant was called when it was present in the “analysis visualization” on Ion Reporter where the *TP53* loss of function variant with a molecular frequency less than 1% was further filtered out if the variant had not been defined as a hotspot mutation.

## 3. Results

### 3.1. Summary of the Run

An average of 22 million reads was mapped to the reference genome per library, and 96% of the mapped reads were on a target relative to the designed bed file. The mean depth of coverage ranged from 64,000× to 91,000×. The uniformity of each library, which is the percentage of amplicons (bases) covered greater than 20% of the mean amplicon (base) coverage, was 99%.

### 3.2. Analytical Sensitivity

The analytical sensitivity of SNVs and indels was evaluated with a varying level of VAF and input using the Multiplex I cfDNA Reference Standard (Table 1).

Molecular coverage depended on the amount of input. A molecular coverage above 2000× was obtained with 20 ng of input, whereas molecular coverages of 1100× and 700× were obtained with 10 and 5 ng of input, respectively. With 20 ng of input, the analytical sensitivities at VAFs of 0.1%, 0.5%, and 1% were 50%, 93.4% and 100%, respectively. At a VAF of 0.1%, the *EGFR* p.E746_A750del variant was detected in a repeated experiment, although other mutations were inconsistently detected below the limit of detection (LOD) level. At a VAF of 0.5%, the *EGFR* L858R mutation was detected only once in duplicate experiments, while other mutations were detected in both. Although two molecular reads of 1752 molecular depth (0.11%) had the variant, the variant was treated as “NOCALL” with the reason of “&VARCOV<3&VARCOV-TGSM<3”. This means that it did not satisfy the detection threshold of a molecular coverage ≥3.

For evaluation of the analytical sensitivity for fusion, we tested libraries having two levels of VAF or copies at 20 ng. As a result, *ROS1* and *RET* fusion transcripts were detected at VAF levels of both 5% and 1%. For CNV, copy number changes of *MET* with 4.5 (2.25-fold change) and 2.5 copies (1.25-fold change) were detected, and their observed fold changes were 1.8 and 1.44, respectively (Table 2).

### 3.3. Analytical Specificity

Analytical specificity was estimated using pooled plasma obtained from healthy subjects aged 30–40 years.

The plasma was tested in seven independent experiments, and 1.0–10 ng of nucleic acid was used. The median molecular depth was 900× (range 583~1600×). As a result, no variant was called except two false positive variants, *KIT* p.V530I and *MAP2K1* p.F129L, from one experiment. The false positive variants were hotspot mutations, and their VAFs were 0.28% and 0.24%, respectively.

### 3.4. Precision

Precision was estimated from the replicated data of the reference material within a run and/or between runs. For precision at a VAF of 1% with 20 ng of input, two replicates within the run and six replicates with independent batches were performed. The overall precision of the true positive variants was 98% (Table 3).

For individual variants, all except *EGFR* p.L858R showed a precision of 100%; *EGFR* p.L858R was detected in 7 of 8 replicates, yielding a precision of 87.5%. Although three molecular reads of 3525 molecular depth (0.09%) had the variant, it was treated as “NOCALL” with the reason of “.&VARCOV-TGSM<3.” This means that it did not satisfy the detection threshold of molecular coverage ≥3 after adjustment using TGSM (TaG Similarity), which is the number of families that supports alleles being falsely generated due to a PCR error or family cloning from sample preparation. Coefficients of variation (CVs) of the observed VAF varied from 16% to 62% according to the variant.

In addition to the verified mutations, the following five variants were called consistently at VAFs of 20~30% across the eight replicated experiments: *BRAF* p.V600E, *CTNNB1* p.S33Y, *EGFR* p.G719S, *MAP2K1* p.Q56P, and *PIK3CA* p.H1047R. According to the manufacturer’s claim, those variants were confirmed in parent cell lines by whole exome sequencing (https://horizondiscovery.com/en/reference-standards/products/~/link.aspx?_id=D2686851191C40738A817A951F90BA82&_z=z, accessed on 15 January 2021).

In addition to the above variants, *FGFR3* amplification was called in 7 of 8 replicates. Each *MET* amplification, *APC* p.S1465fs, *FBXW7* p.R465C, and *HRAS* p.G12V were called once.

At a VAF of 0.5% with 20 ng, only the repeatability (intra-run) of true positive variants was estimated from the reference material; the overall repeatability was 94% as the *EGFR* p.L858R variant was detected only once among the two replicates (Table 1).

### 3.5. Comparison Between Cobas-EGFR and Pan-Cancer Cell-Free Assay

When using the reference material at VAFs of 0.5 and 1.0% with 20 ng of input, the Pan-Cancer Cell-Free Assay and Cobas-EGFR showed 100% detection (Table 4).

At a VAF of 0.1%, the Pan-Cancer Cell-Free Assay detected two of four variants, whereas Cobas-EGFR detected one of four.

In clinical samples, the amount of input ranged from 0.7 to 53 ng with a molecular depth of 1053~3806×. Across the four libraries from eight clinical samples, all *EGFR*-mutations detected by Cobas-EGFR were also detected by the Pan-Cancer Cell-Free Assay, showing 100% concordance (Table 5). The lowest VAF was 0.1% for the p.E746_A750del mutation.

## 4. Discussion

In this paper, we evaluated the analytical validity of the Pan-Cancer Cell-Free Assay on an S5 platform for detecting cancer-associated mutations. As analytical performance depends on the amount of input nucleic acid, as well as VAF, we planned the evaluation protocol with variable VAF, and the amount of input based on reference materials.

It has been suggested that a 0.1% of LOD is required for tumor liquid biopsy, whereas an LOD less than 0.01% is required for early cancer detection [13]. In the present study, the analytical sensitivities were estimated to be 50%, 94%, and 100% at VAFs of 0.1%, 0.5%, and 1% with 20 ng of input, respectively. If limited numbers of mutant molecules are present, the accurate and reliable detection of mutation with a VAF of 0.1% is challenging due to the stochastic sampling issue [14]. This issue is a problem for the Pan-Cancer Cell-Free Assay, as well as others. Previous studies that have included patients with various cancer types showed a mean VAF of stage III/IV greater than 1% [15] and a VAF for second quartiles of 1.3% in metastatic cancers [16], indicating that this assay is suitable for advanced cancer patients.

Detection sensitivity differed by mutation. Though massively parallel sequencing (MPS) is less sensitive for the detection of indels than for SNVs [17,18], the detection of the *EGFR* p.E746_A750del mutation in this study was better than that of other *EGFR* mutations and was consistently detected in duplicated experiments at a VAF of 0.1%. The detection of the *EGFR* p.L858R mutation was less sensitive as it was detected only once in two duplicated experiments at a VAF of 0.5%. In addition, the reproducibility of detection of the *EGFR* p.L858R mutation at a VAF of 1% was relatively low as one of six additional experiments failed to detect the mutation despite adequate coverage of that position (3525 molecular reads). *EGFR* exon 19 deletion and L858R are the first and second most common mutations found in Asian lung cancer patients, respectively [19]. This demonstrates the importance of the awareness of issues related to the sensitivity and/or precision for each clinically important mutation.

In the comparison test using the reference material, the Pan-Cancer Cell-Free Assay and Cobas EGFR assay showed comparable results. Both assays showed 100% detection of the *EGFR* mutations at VAFs of 0.5% and 1% with 20 ng of input. Both assays showed decreased sensitivity at a VAF of 0.1% with the same input. However, the Pan-Cancer Cell-Free assay appeared to be slightly more sensitive than the Cobas EGFR assay, with 50% and 25% detection of the variants, respectively. In a previous study, the Cobas EGFR assay showed 50% detection at a VAF of 0.1%, while the Oncomine Lung cfDNA assay detected all variants [20]. The difference in the detection rate between the previous and current studies might be due to the different amounts of input nucleic acid (66 vs. 20 ng).

The performance of the test can be different even with the same platform or the same assay. Thus, reporting from various studies by independent groups may help to determine the performance of the assay. The previous study using the Oncomine Lung Cell-Free DNA Assay, the same platform with the different assay, demonstrated a sensitivity of 81% with <20 ng of cfDNA at a VAF of 0.1%, whereas our data showed a sensitivity of 50% under the same condition [21]. In the report by the manufacturer, the same assay in the present study showed a sensitivity of 80% at a VAF of 0.1% for SNV [22]. In the present study, most of the analytical performance tests were conducted using reference materials and clinical samples containing only *EGFR* mutations. Other clinically important mutations including fusion or CNV were not validated by clinical samples. As the performance with clinical samples may not be the same as that with reference materials, further studies would be required for analytical validity of this panel. Although analytical validity has been rarely reported for this assay, clinical validity has been reported in endometrial cancer, brain tumor, and colorectal cancer [23,24,25]. The clinical validity of various cancer types remains to be determined.

## 5. Conclusions

The Pan-Cancer Cell-Free Assay can be applied to detect *EGFR* mutations in advanced lung cancer patients, although follow-up studies will be needed to evaluate the analytical validity for other types of genes and aberrations using clinical samples. As sensitivity varies according to variant, the clinical laboratory should evaluate and understand the performance characteristics of the assay prior to its adoption as a clinical test.

## Figures and Tables

**Table 1:** Analytical sensitivity using the reference material.

Gene	Variant	Variant Allele Frequency (%) According to Input (ng)							
		0.1%				0.5%		1%	
		5 ng	10 ng	20 ng		20 ng		20 ng	
*EGFR*	p.L858R						0.7	0.94	0.62
	p.E746_A750del			0.26	0.25	0.55	0.59	1.82	1.44
	p.T790M			0.2		0.44	0.52	1.08	1.31
	p.A767_V769dup		0.16		0.08	0.32	0.32	1.07	0.67
*KRAS*	p.G12D		0.53	0.2		0.37	0.68	1.22	1.25
*NRAS*	p.Q61K				0.18	1.27	0.86	1.99	1.36
	p.A59T					0.72	0.55	1.35	2.41
*PIK3CA*	p.E545K			0.16	0.32	0.41	0.97	1.34	1.44
Molecular coverage		688×	1139×	2091×		2115×		2017×	
Analytical sensitivity		0% (0/8)	25% (2/8)	50% (8/16)		94% (15/16)		100% (16/16)	

**Table 2:** Analytical sensitivity for fusion and CNV using the reference material.

**Variant (Fusion)**	**VAF 1%**			**VAF 5%**		
	**Result**	**Mol. Cov. for Fusion**	**Read** **Cov. for Fusion**	**Result**	**Mol. Cov. for Fusion**	**Read** **Cov. for Fusion**
SLC34A2–ROS1	Detected	31	1473	Detected	127	4850
CCDC6–RET	Detected	12	510	Detected	32	1357
**Variant (CNV)**	**2.5 Copies (CN Ratio of 1.25)**			**4.5 Copies (CN Ratio of 2.25)**		
	**Result**	**Observed** **CN Ratio**	**MAPD**	**Result**	**Observed CN Ratio**	**MAPD**
MET amplification	Detected	1.44	0.233	Detected	1.8	0.24

Abbreviations: CN, copy number; CNV, copy number variation; MAPD, median of the absolute values of all pairwise differences; VAF, variant allele frequency.

**Table 3:** Precision at a VAF of 1% with 20 ng of reference material when testing duplicates within the same batch and six replicates from independent batches.

Gene	Variants	Expected VAF (%)	Mean and SD of Observed VAF (%)	CV (%)	Detection Rate
*EGFR*	p.L858R	1.0	0.99 ± 0.23	23	87.5% (7/8)
	p.E746_A750del		1.38 ± 0.37	27	100% (8/8)
	p.T790M		1.00 ± 0.37	37	100% (8/8)
	p.A767_V769dup		0.94 ± 0.29	31	100% (8/8)
*KRAS*	p.G12D	1.3	1.29 ± 0.32	25	100% (8/8)
*NRAS*	p.Q61K		1.43 ± 0.23	16	100% (8/8)
	p.A59T		1.66 ± 0.44	27	100% (8/8)
*PIK3CA*	p.E545K		0.95 ± 0.58	61	100% (8/8)

Abbreviations: CV, coefficient of variation; SD, standard deviation; VAF, variant allele frequency.

**Table 4:** Comparison between Oncomine Pan-Cancer Cell-Free Assay and Cobas-EGFR assay for detection of *EGFR* mutations using the reference material.

Variant	0.1%		0.5%		1%	
	Pan-Cancer	Cobas	Pan-Cancer	Cobas	Pan-Cancer	Cobas
	VAF (%)	SQI	VAF (%)	SQI	VAF (%)	SQI
p.E746_A750del	0.26	11.36	0.59	12.92	1.82	14.11
p.A767_V769dup	ND	ND	0.32	2.72	1.07	3.1
p.T790M	0.2	ND	0.52	8.75	1.08	9.42
p.L858R	ND	ND	0.7	8.2	0.94	8.5

Abbreviations: SQI, semi-quantitative index; VAF, variant allele frequency.

**Table 5:** Comparison between Pan-Cancer Cell-Free Assay and Cobas-EGFR assay from samples with *EGFR* mutation.

Library No. *	Variant	Cobas-EGFR Assay	Pan-Cancer Assay		
		SQI Value	Molecular Depth	Molecular Count	Observed VAF (%)
1	p.E746_A750del	12.04	3144	3	0.1
	p.E746_A750del	11.18			
2	p.L858R	6.66	2110	210	9.95
	p.T790M	11.61	886	83	9.37
3	p.L858R	7.01	1540	32	2.08
	p.L858R	7.87			
4	p.L858R	5.01	1770	5	0.28
	p.L858R	5.99			

* Each library was prepared by mixing plasma from two clinical samples. Abbreviations: SQI, semi-quantitative index; VAF, variant allele frequency.
